# An international and interdisciplinary framework for nature prescribing in healthcare: A modified Delphi study

**DOI:** 10.1371/journal.pgph.0006361

**Published:** 2026-04-30

**Authors:** Nicole A. Struthers, Aleksandra A. Zecevic, Jessica Stanhope, Martin F. Breed, Lesley Gittings, Anna Gunz, Amy Wagenfeld, Trevor Birmingham, Anita Kothari, Filip Maric

**Affiliations:** 1 Health and Rehabilitation Sciences, Faculty of Health Sciences, University of Western Ontario, London, Ontario, Canada; 2 School of Health Studies, Faculty of Health Sciences, University of Western Ontario, London, Ontario, Canada; 3 Rheumatology Unit, The Queen Elizabeth Hospital, Adelaide, South Australia, Australia; 4 School of Public Health, The University of Adelaide, Adelaide, South Australia, Australia; 5 School of Allied Health Science and Practice, The University of Adelaide, Adelaide, South Australia, Australia; 6 Environment Institute, The University of Adelaide, Adelaide, South Australia, Australia; 7 College of Science and Engineering, Flinders University, Adelaide, South Australia, Australia; 8 Centre for Social Science Research, University of Cape Town, Cape Town, South Africa; 9 Children’s Health Research Institute, London, Ontario, Canada; 10 Department of Paediatrics, Schulich School of Medicine and Dentistry, University of Western Ontario, London, Ontario, Canada; 11 Department of Landscape Architecture, University of Washington, Seattle, Washington, United States of America; 12 Bone and Joint Institute, University of Western Ontario, London Health Sciences Centre-University Hospital, London, Ontario, Canada; 13 School of Physical Therapy, Faculty of Health Sciences, University of Western Ontario, London, Ontario, Canada; 14 Department of Health and Care Sciences, Faculty of Health Sciences, UiT The Arctic University of Norway, Tromsø, Norway; University of Oslo Faculty of Medicine: Universitetet i Oslo Det medisinske fakultet, NORWAY

## Abstract

Nature prescriptions allow healthcare providers to recommend nature connection and exposure for health and well-being benefits. However, an accepted interdisciplinary shared definition of nature prescription is currently lacking, as are frameworks to guide the use of nature prescription in healthcare. To help fill this knowledge gap, we used an international modified Delphi technique to develop a framework for nature prescribing in healthcare by interdisciplinary experts. Round 1 consisted of virtual focus groups with an expert panel to generate a preliminary framework and framework statements through a qualitative approach, followed by Round 2 to establish agreement, set a priori as ≥75%, on statements to be included in the presented framework. Eight expert panellists participated in the consensus building process, developing and agreeing on 146 statements. The framework consists of: (1) Defining nature prescriptions; (2) Human benefits and risks; (3) Environment benefits and risks; (4) Society and Culture contextual factors and perspectives, acknowledgement of Indigenous knowledges, and equity; (5) Planetary health as a positive feedback loop between the benefits for people and environments; (6) Future directions to aid moving knowledge and implementation of nature prescribing forward. We report an overview of the benefits and risks of nature prescriptions, their potential planetary health impacts, and how they can be equity-driven by incorporating social and cultural differences. This international and interdisciplinary framework for nature prescribing in healthcare offers itself as a guide for the utilization of nature prescriptions in healthcare, and proposes recommendations for research, education, and clinical practice.

## Introduction

Prescribing connection and engagement with nature is an emerging area of interest situated within the human-nature relationship, sustainable healthcare, and planetary health [1,2]. Sustainable healthcare is a healthcare approach that aligns with planetary health, and includes health professionals and systems emphasizing environmental accountability and stewardship to take care of people and the planet [3]. Health systems can contribute to planetary health by working towards net-zero healthcare emissions, reducing the demand for health services, matching the supply of services to the demand, and reducing the emissions from the supply of health services [4]. Furthermore, individual actors within health systems can implement environmentally sustainable health services as alternatives to services with higher carbon emissions [5,6], as well as incorporating approaches to experiencing the beneficial health effects of the human-nature relationship [1].

Research on the links between human health and nature has expanded our understanding of the benefits of connection with nature and greenspace exposure [7–9], as potential contributors to sustainable healthcare and planetary health. Greenspace refers to undeveloped land with natural vegetation, parks and publicly accessible vegetation, and open land or forest [10]. The term ‘greenspace’ has been used both synonymously with ‘nature’ and in reference to urban vegetation [10]. Biodiversity of greenspaces contribute to human health and well-being, both positively and negatively, through biological, psychological, social, physical activity, and harm-causing pathways, with a moderate-high level of evidence supporting broader nature pathways [11].

Benefits of greenspace exposure and contact with nature have been found for physical health indicators, both in general and populations with diverse health conditions. Positive impacts have been noted on cardiometabolic health [12–14], biomarkers of inflammation [12,13], self-reported health [14], pain [13,15], and physical activity [12,13,16]. Specific to health conditions, nature-based interventions may have a beneficial effect on condition-specific outcomes and health indicators, including cardiovascular risk factors and body composition [17]. Mental health benefits of greenspace exposure have been reported on stress, anxiety, depression, loneliness [12,13], happiness [12], and decreased prevalence of psychopathology [18]. The synergistic benefits of exercise in greenspaces may outweigh the benefits of indoor exercise alone [19,20], regarding physical, mental, and social well-being [21–23]. The availability of greenspace can facilitate social engagement and physical activity [20,21,24], and having diversity in nature experiences has been shown to positively correlate with life satisfaction [25]. Nature-based interventions are valuable in health management, especially for people living with chronic health conditions, for the variety of reported benefits of nature-based interventions, greenspace exposure, and exercising in green spaces. Despite the health and wellbeing benefits of connecting with nature, these benefits are inequitably distributed, due to environmental racism, that is: “environmental policies, practices, or directives that disproportionately disadvantage individuals, groups, or communities (intentionally or unintentionally) based on race or colour” [26]. These include pollution and environmental hazards disproportionately occurring in racialized neighbourhoods and the historical and ongoing dispossession of Indigenous Peoples from Land [27].

Given the diversity of health and well-being benefits associated with greenspaces and nature, coupled with the importance of integrating sustainable healthcare strategies, the use of nature-based interventions in healthcare should be considered. As one form of nature-based intervention, nature prescriptions allow healthcare providers to recommend that their patients and clients engage in nature connection and exposure to reap a variety of health benefits [28–30]. Nature prescription programs are gaining momentum in the Global North and Western health systems, including programs established in the United Kingdom [31,32], Scotland [33], the United States [29,34–36], and Canada [37,38]. This momentum can further be observed within research literature, where the progression towards structured guidance on nature prescribing can be seen in the emergence of a nature prescribing framework for the Australian healthcare context [39].

Relationships between nature and human health have been well-established in traditional medicines, practices, and ways of knowing [40]. Indigenous Peoples’ knowledges and approaches to health have long reflected principles of interconnectedness [41,42], such as Land-based healing, a practice Indigenous to Turtle Island centring relationships with the Land within the healing process [43,44]. Shinrin-yoku, coined in 1982 [45], is a practice from Japan that involves immersing oneself in a natural environment [45,46]. Ancient healing practices and traditional medicine throughout history and cultures have included the natural world as a core tenant of healing, medical treatments, and world views since time immemorial [40,47]. Throughout the twentieth century, Western hospital architecture reflected the value of rehabilitation gardens and open-air therapy, such as sanitoria centred around sun terraces between 1900 and 1940 and an emergence of healing gardens in outpatient and day treatment facilities [48]. However, within modern-day Western medicine, the practice of prescribing nature in healthcare settings is viewed as being in its infancy [49] and unorthodox [50]. To support nature prescribing in healthcare, a framework is needed to provide knowledge to users, facilitate wide-scale implementation [51], and provide a shared definition of nature prescriptions to support harmony within vocabulary [50].

The purpose of this international and interdisciplinary modified Delphi study was to develop a framework for nature prescribing in healthcare that summarises the collective knowledge and opinions of experts and provides direction for further development and implementation in research, education, and clinical practice. We address the identified needs for (1) a shared definition of nature prescription [29,50], and (2) a descriptive framework to guide the use of nature prescribing in healthcare [51,52]. Furthermore, we sought to answer two broad research questions: (1) How can the utilization of nature prescriptions address personal, social, environmental, and global burdens of health and healthcare? and (2) What is the direction for future research on and utilization of nature prescriptions? This study was aimed towards healthcare and health-related professions; however, the findings may be of value to environmental science, urban planning, policymakers, and other interdisciplinary fields.

## Materials and methods

### Ethics statement

The study protocol was approved by the University of Western Ontario’s Health Sciences Research Ethics Board (REB # 123288). Formal consent was obtained via electronic written consent.

### Positionality statement

The author team holds various roles and identities. Authors include the Principal Investigator, the core research team in London, Ontario, Canada, and participants of the expert panel representing three continents and four countries. We hold diverse cultural and gender identities, and are people of different ages, abilities, and career stages.

### Study design

We report on findings of a two-round modified Delphi study aiming to develop a framework for use of nature prescribing in healthcare. The Delphi technique is an iterative survey process to collect expert input with the aim of achieving expert consensus on a topic that is in the explorative and theoretical phase [53,54] or where there is incomplete evidence or knowledge [55,56]. Unlike the traditional Delphi method, the modified Delphi technique allows for expert interaction during the process and the generation of items selected by the expert panel and extracted from existing literature [53,57–59].

Round 1 utilized focus groups with expert panellists to generate statements through interactive discussions, a modification to the traditional anonymous Delphi survey technique [53]. Round 2 consisted of a survey to determine panellist agreement on the inclusion of statements generated in Round 1. This modified Delphi includes the adaptations of using both qualitative and quantitative data as well as different data collection methods for each round [53].

### Setting

This study was conducted in partnership with the Environmental Physiotherapy Association (https://environmentalphysio.com), the first international network of academics, clinicians, organizations, and students seeking to advance environmental perspectives across physical therapy research, practice, and education [60]. This cross-sectoral network optimizes the potential for social and intellectual impact of the present study through knowledge co-creation and mobilization between academic and non-academic audiences. As such, the founder and executive chair of the Environmental Physiotherapy Association (FM) in Norway and the research group at the Western University (NS & AZ) in Canada, formed the steering group to lead this modified Delphi study.

### Participant selection and recruitment

We aimed to achieve a panel of 10–15 participants, based on typical sample sizes for the Delphi method in health research [61,62] and recommendations for a heterogenous expert panel coming from various disciplines [56,63]. A preliminary list of potential expert panel members (n = 30) from around the world was developed and discussed by the steering group. Other potential expert panel members were identified through reviewing literature on nature prescriptions and other nature-based interventions, with additional suggestions provided by the steering group. Inclusion criteria for the expert panel were: (1) relevant expertise and experience, either in research or practice; and (2) able to speak, read and write in English. Relevant expertise and experience included publishing or conducting research on nature prescriptions and related areas, presenting on nature and health research in international conferences, or being a member of the Environmental Physiotherapy Association’s Executive Committee. Potential panellists identified by the steering group who met inclusion criteria (n = 24) were contacted by email and invited to participate in the study. Informed consent was received from each panellist who agreed to participate (n = 11), via written electronic signature, after the purpose of the study and their expected contributions were explained, including the opportunity for co-authorship. Three participants were lost to follow-up, resulting in full participation of eight experts in this study. The participant recruitment period took place between November 7, 2023, and February 2, 2024.

### Round 1

#### Data collection.

Expert panel members were asked to complete an online demographic characteristics survey administered through Qualtrics (Qualtrics, Provo, UT, http://www.qualtrics.com/). A focus group guide, consisting of 19 open-ended questions grouped in six broad areas of inquiry, was developed and refined by the steering group’s (NS, AZ, FM) expertise and informed by gaps in literature. The guide was separated into three topically separate focus group sessions: (1) Broad definitions of terms, concepts and connections; (2) Addressing the environmental burden of health management and healthcare, integrating sociocultural considerations, moving towards planetary health, and future research; and (3) Logistics of implementing into clinical education and practice – the who, what, when, where, why, and how. Virtual focus group sessions lasting 60–90 minutes each were scheduled to ensure sufficient yet timely discussion whilst limiting participant fatigue [64], and were recorded and transcribed using Zoom (Version 5.17.1). To accommodate for time zones and schedule compatibility, each topic session was held twice for a total of six virtual discussions. Written responses were accepted from panellists unable to attend a specific session.

#### Data analysis.

For demographic variables, the count (n) and proportion of the total sample (%) were calculated. Transcripts and written responses were uploaded into NVivo (Version 14) to store, code, and manage the data. A thematic analysis approach [65] was used to analyse and translate the panellists’ responses into candidate statements. First, transcripts and written responses of the 19 questions were read and re-read by the first author (NS) and a research assistant to gain deep familiarity with the data. The data within each focus group question underwent open coding to identify relevant text segments. The first author and a research assistant coded 30% (n = 6) of the question transcripts independently, compared the developed codebooks, and discussed differences until agreement was reached. After initial coding was complete, preliminary themes and sub-themes were identified, and subsequently reviewed, modified, and further developed in collaboration with a senior author (AZ). The first author re-read the data using the themes and sub-themes as a guide, to ensure all relevant text segments were captured. Data that did not fit within any theme or sub-theme were coded as “Other”. Candidate statements were developed from the coding of relevant text segments and organised within the themes and sub-themes. The first and second authors (NS & AZ) independently reviewed the candidate statements alongside the transcripts to ensure accuracy and comprehensiveness, followed by a discussion to check for agreement. All partial data, collected from participants up to declaration of being lost-to-follow-up, were included in analysis.

### Round 2

#### Data collection.

The themes and sub-themes identified in Round 1 were used to develop matrix tables, where each candidate statement was presented with three response categories [66,67] to indicate agreement, and a free-text option for panellists to provide any modifications or additional statements. A 3-point scale was selected for simplicity during the process of determining which statements should or should not be included in the framework. The list of candidate statements was sent to the same expert panellists with a request to rate their agreement for inclusion of each statement through a Qualtrics survey (Qualtrics, Provo, UT, http://www.qualtrics.com/). Panellists provided their agreement according to the following criteria: (1) Disagree: Disagreeing on the inclusion of the statement in the framework; (2) Agree: Agreeing on the inclusion of the statement as-is in the framework; and (3) Agree with modification: Modifications must be made to agree with the inclusion of the statement in the framework.

#### Data analysis.

Percentage agreement was used to analyse responses [68]. Based on previous Delphi studies [69], agreement was set *a priori* at ≥75% for statements to be included in the framework, summative of ‘Agree’ and ‘Agree with modification’. Additional statements were included if modifications substantially altered the meaning of the original statement or if a new statement was proposed.

## Results

**Fig 1:**
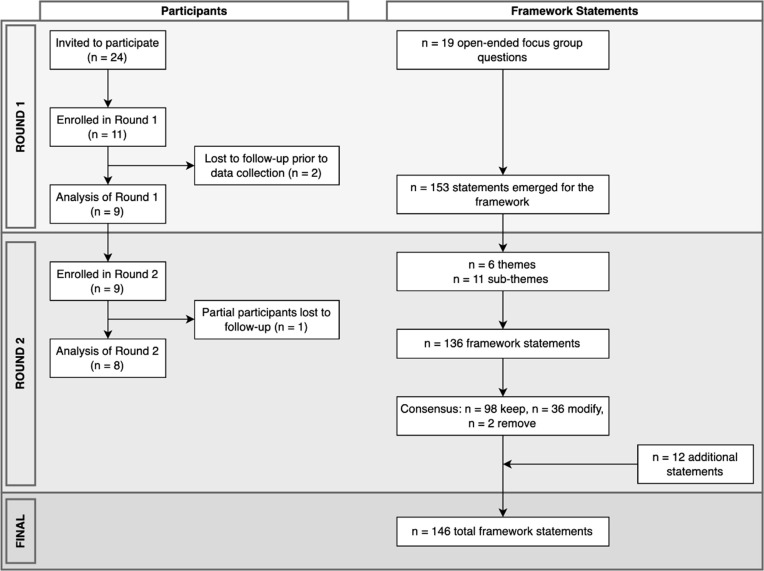
Flow of expert panel participants and framework statements throughout Round 1 and Round 2.

The flow of study participants alongside the evolution of the framework statements is presented in Fig 1. The demographic characteristics of the expert panel are presented in full in S1 Table. Of the eight participants, most were female (75%), mid-career (75%), highly educated (75% with PhDs), and lived in large urban population centres (63%) of English-speaking countries (Australia, Canada, Norway, Sweden, United States of America) in the Global North (100%). Indigenous experts and experts located in the Global South were invited; however, they were not available to participate in this study at the time of recruitment. Participants were interdisciplinary researchers at intersections of the following disciplines: physiotherapy, medicine, occupational therapy, epidemiology, planetary health, geography, restoration ecology, ecosystem health, social aspects of health, and landscape architecture, and civil engineering. Round 1 generated six themes, 11 sub-themes, and 136 candidate statements to be reviewed by the panel in Round 2, where the agreed (n = 98), modified (n = 36), and additional (n = 12) statements were included in the framework. The individual agreement statistics and unmodified statements can be found in S1 File (1 & 2).

### A framework for nature prescribing in healthcare

**Fig 2:**
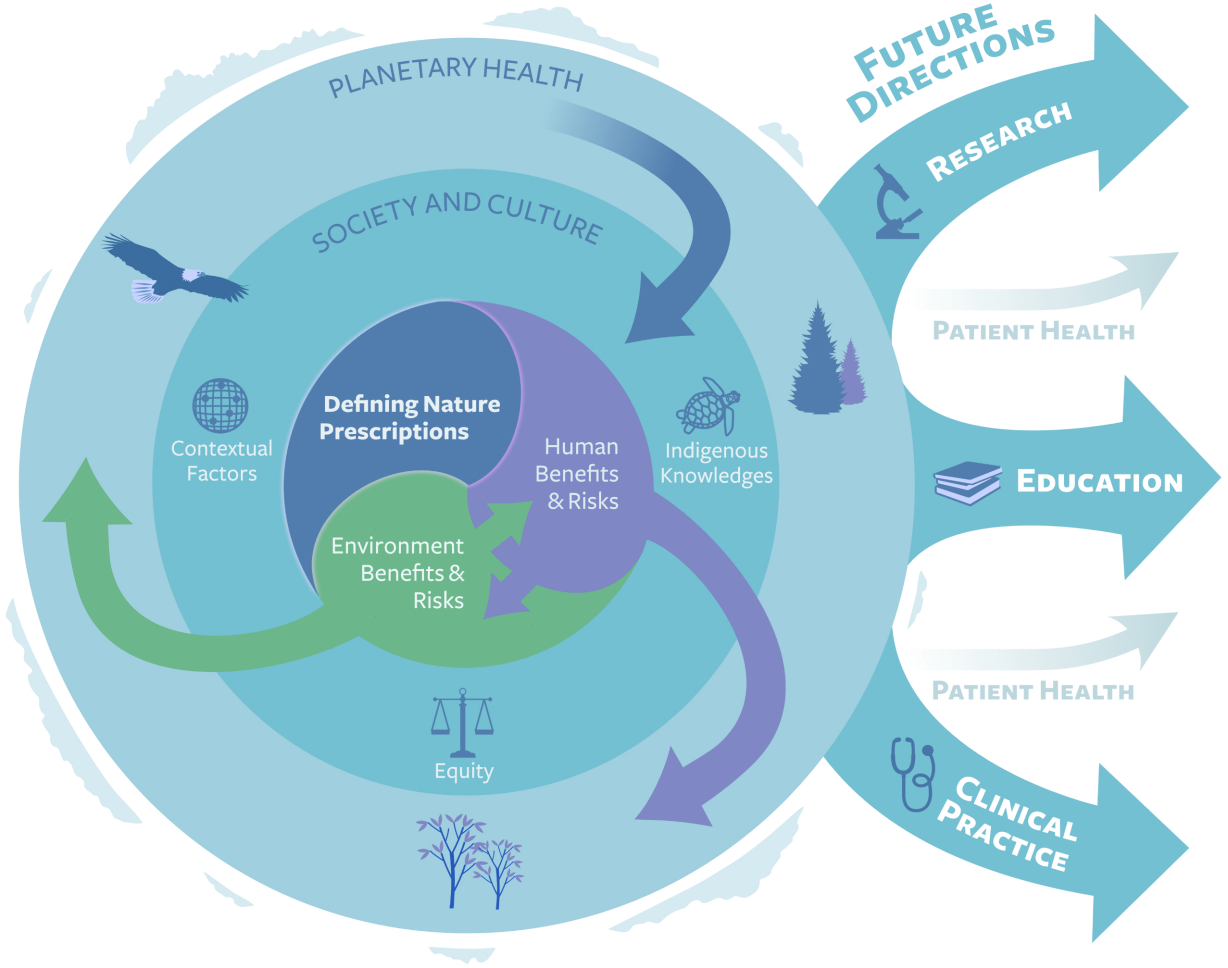
Full schematic of the international and interdisciplinary framework for nature prescribing in healthcare. *Note.* To acknowledge the land and territories where this research was conceived and conducted, a turtle (Mikinaak/Mshiikenh) was chosen to acknowledge Turtle Island (Canada) and the original guardians and care takers of the Land from time in memorial; Indigenous peoples of First Nation, Métis, and Inuit descent. Haudenosaunee and Anishinaabe creation stories place the turtle as the start of the development of the world we live in, which was created on the turtle’s back. The turtle recognises each direction of Canadian nations and clan systems, including the four nautical directions, the sky, Mother Earth, and the water. The eagle (Migizi) was chosen as they fly highest to the Creator (Gchi Manidoo) and serve as teachers who carry lessons to be passed down through generations. We recognise that across this Land, Indigenous peoples have endured historical and ongoing oppression and inequities resulting from the colonialist systems, ideologies, and worldviews that continue to be perpetuated to this day. We acknowledge the contributions of Waabishki Mukwa Kwe, an Anishinaabe Indigenous consultant, who guided the imagery, language, and teachings of this figure.

The emerging framework for nature prescribing in healthcare reflected six themes, 11 sub-themes, and 146 statements, with connections between the framework sections (Fig 2). The major themes of considerations of nature prescribing in healthcare were: Defining Nature Prescriptions (n = 11 statements); Human Benefits and Risks (n = 17 statements); Environment Benefits and Risks (n = 14 statements); Society and Culture (n = 7 statements); Planetary Health (n = 13 statements); and Future Directions for Research (n = 23 statements), Education (n = 21 statements), and Clinical Practice (n = 40 statements). Sub-themes were related to the characteristics that define nature prescriptions, the potential benefits and risks, factors to consider, and how research, education, and practice could proceed.

### Defining nature prescriptions

How “nature” and “prescription” are defined influences the anticipated outcomes on people and the environment, all of which are understood by each person through their unique social and cultural lens. Derived from the framework statements, nature prescriptions can be defined as written or verbal instructions to engage with nature as the context for an intervention that includes guidance on dosage and activities (Table 1). Nature, as the context for an intervention, may involve areas maintained for their natural biotic and abiotic characteristics, biodiverse spaces and native ecosystems as non-anthropogenic contexts, human-modified environments, and indoor or outdoor environments that include components of nature (Table 1). Features of what “nature” and “prescription” represent are provided along with examples, such as components of nature (Statement 6). Nature prescriptions are transdisciplinary; hence, the defining features must reflect this adaptability. Developing one singular, absolute definition for both “nature” and “prescription” would be futile, if not impossible. The framework suggests characteristics of nature prescriptions; however, the characteristics must resonate with a person’s beliefs and values, which are influenced by their context in the world and what they deem to be true.

**Table 1:** Statements defining nature prescriptions.

Section	#	Statement
**Defining Nature Prescriptions**		
*Nature is…*		
	1	Outdoor natural environments
	2	Spaces that largely include native ecosystems and biodiversity
	3	Human-modified environments (e.g., gardens, parks, farms, lakes)
	4	Indoor green spaces (e.g., indoor gardens, nature scenes)
	5	Areas managed for their natural values/assets^a^
	6	Components of nature, both indoors and outdoors (e.g., rooftop gardens, indoor potted plants, animal environments [paludariums, aquariums, zoos])^a^
*Prescription is…*		
	7	Written instructions
	8	Verbal instructions
	9	Guidance on dosage (e.g., frequency, intensity, duration, timing)
	10	Guidance on activities (e.g., specific nature locations, potential incentives)
	11	An instruction to utilize nature as the context for the intervention

*Note.* ^a^ = Statement suggested as an addition during Round 2.

### Benefits and risks to humans and the environment

The benefits and risks of nature prescriptions to people and the environment are numerous and diverse (Table 2). Benefits of nature prescribing for people and the environment are plentiful, including those on human health (Statements 12, 13, 16, 17), human-environment relationships (Statements 14, 18, 29, 30), and the health of environments and ecosystems (Statements 31, 33–35). Both the benefits and risks can vary depending on how nature and prescription are defined. For instance, exposure to biodiverse environments (Statement 14) may not be a benefit experienced by people if nature is defined as components of nature, both indoors and outdoors (Statement 6). Components of nature alone might not be representative of biodiverse environments; hence, the benefit of exposure to biodiversity may not hold true in this circumstance. Conservative definitions of nature may lower the risk of harm to people and the environment. If nature was defined only as being spaces that largely include native ecosystems and biodiversity (Statement 2), increased human engagement with such nature spaces may lead to ecosystem degradation and biodiversity loss (Statement 36). These nature spaces may not have undergone development to include anthropocentric features; thus, people using these spaces to participate in nature prescriptions may face risks. For example, injury (Statement 21) from tripping on unlevel ground and plants, environmental exposures (Statement 23) including vector-borne illnesses, allergens, climatic factors (e.g., sun), and animals (e.g., venomous snakes, bears), and a lack of physical supports in outdoor spaces (Statement 25) to assist with human needs, such as the availability of safe rest areas and toilets which provide interoceptive support.

**Table 2:** Statements describing the benefits and risks of nature prescriptions to humans and environments.

Section	#	Statement
**Human Benefits and Risks**		
*Potential benefits of nature prescriptions to humans are…*		
	12	Health condition-specific outcomes (e.g., reduced pain, improved quality of life, improved motor control)
	13	Psychological (e.g., reduced anxiety and stress, improved confidence)
	14	Exposure to biodiverse environments (e.g., immune regulation from exposure to microbiota)
	15	Social (e.g., reduced isolation, improved connection, group-based outdoor activities)
	16	Increased physical activity (e.g., use of nature spaces conducive to exercise)
	17	Improvements in comorbidities (e.g., simultaneous cardiovascular and mental health benefits)
	18	Improved connection and relationship with nature
	19	Exposure to increased challenges (e.g., unpredictable outdoor surfaces, risk exposure)
	20	Increased capacity to learn and retain information (e.g., becoming more familiar with plants, learning to grow own food)^a^
*Potential risks of nature prescriptions to humans are…*		
	21	Injury (e.g., tripping, falls)
	22	Inclement weather (e.g., ice, extreme heat)
	23	Environmental exposure (e.g., sun, soil-, vector- or water-borne pathogens, allergens, air quality)
	24	Damage to assistive devices
	25	Lack of physical supports in outdoor spaces (e.g., benches, rest areas, toilets)
	26	Psychosocial harm (e.g., feeling vulnerable, patronised, or disrespected)
	27	Unsafe environments (e.g., outdoor violence, poorly maintained infrastructure, neighbourhood crime or violence)
	28	Financial risk (e.g., direct and indirect costs of participation)
**Environment Benefits and Risks**		
*The environment can benefit from nature prescriptions through…*		
	29	Promoting pro-environmental behaviours (e.g., advocacy, stewardship)
	30	Increasing the perceived value of nature
	31	Environmental conservation and restoration (e.g., planting native species, removing weeds)
	32	Increased funding (e.g., civic budget allocations, non-governmental funding, conservation funding)
	33	Promoting the creation of naturalized areas (e.g., re-establish populations of native plants through planting native species)
	34	The inclusion of activities that support the health of nature, where appropriate^a^
	35	Promoting the ecological integrity of areas (e.g., areas with native plants and animals)^a^
*Nature prescriptions may pose a risk to the environment through…*		
	36	Ecosystem degradation and biodiversity loss (e.g., from increased use of nature directly and indirectly)
	37	Destruction of the natural environment (e.g., urbanization, concrete paths, toilets, recreational spaces)
	38	Introduction of novel, non-native species into an ecosystem
	39	Impacting native animals (e.g., human-animal conflict, disrupting animal behaviours)
	40	Increased littering and pollution
	41	Foot traffic damage to ecosystems (e.g., trampled plants, disruptive noise, damage caused by assistive devices)
	42	Reifying the idea of nature as a tool for human use and extraction^a^

*Note.* ^a^ = Statement suggested as an addition during Round 2.

### Society and culture

Societal and cultural contexts are the mesh in which the definitions, benefits, and risks of nature prescriptions reside (Table 3). They are the context in which nature prescriptions can be understood by each person and they determine how we see or understand health. The diversity of knowledge, traditions, and connection with nature is foundational to understanding nature prescriptions and human health. The findings from this modified Delphi study emphasize the importance of acknowledging Indigenous knowledges and attending to sociocultural factors that support nature prescribing. These align with Redvers and colleagues’ (2022) Indigenous Determinants of Planetary Health, which document how Indigenous Peoples’ self-determination and Land-tenure rights are essential to planetary health [70]. Indigenous Peoples around the world have a connection and relationship with nature, often referred to as Land and Country, that does not inherently exist in Western cultures, perspectives, and scientific knowledge systems, as described in detail by Redvers and colleagues [70]. Honouring and centring Indigenous perspectives in nature prescribing can foster holistic benefits when the inherent power differentials within colonial healthcare are addressed through healthcare professionals’ cultural humility, positionality, and purposefully being attuned [71] to the directives of Indigenous Peoples (Statement 44 & 45). The barriers and facilitators to spending time in nature spaces are context specific (Statement 48). By including social and cultural considerations in nature prescribing, participation and adherence can be fostered by using context-specific definitions, reducing context-specific harms and barriers, and promoting context-specific facilitators and benefits to people, the environment, and socio-cultural contexts in which people reside (Statement 46). The lived experiences, knowledge, and context in the world of each person engaging in nature prescriptions, such as the recipient and provider, is imparted on how nature prescriptions are defined and experienced.

**Table 3:** Statements encompassing societal and cultural considerations.

Section	#	Statement
**Society and Culture**		
*Nature prescriptions can incorporate sociocultural considerations through…*		
	43	Co-designing with patients/clients to allow inclusion of their social and cultural contexts, worldviews, and beliefs
	44	Acknowledging and promoting Indigenous knowledge of nature and healing (where appropriate)
	45	Allowing Indigenous knowledge and approaches to guide development (where appropriate)
	46	Attending to sociocultural differences to foster uptake and adherence of nature prescription activities
	47	Person-centred care (e.g., not making assumptions about beliefs and connections to culture)
	48	Incorporating culturally relevant barriers, concerns, and support systems
	49	Taking an equitable lens, allowing integration of societal and cultural contexts

### Planetary health

The planetary health section of the framework presents our perspectives of the maximum potential of nature prescriptions in healthcare (Table 4). If eligible health systems, healthcare institutions, prescribers, and patients or clients adopted nature prescriptions, the benefits of nature prescriptions on people and the environment could contribute to achieving planetary health. The interconnected benefits to people and the environment can contribute to planetary health through a positive feedback loop. Nature prescriptions can include environmentally beneficial activities that simultaneously provide benefits to the person completing the behaviour. The positive experiences feed into a person’s willingness to continue engaging with their nature prescription. If this scenario were to occur for most nature prescription recipients, the surplus benefits to people and the environment might foster improvements in planetary health. As part of low-carbon healthcare, nature prescriptions can promote planetary health through, but not limited to, substituting manufactured healthcare or training equipment with nature-based alternatives (Statement 60), such as using sand or uneven ground instead of foam pads for balance and lower extremity proprioceptive exercises, and being an environmentally friendly intervention (Statement 62) as compared to pharmaceutical or surgical interventions. Additionally, the human health benefits of nature prescriptions may reduce the demand on ecologically damaging treatment options (Statement 61), such as consuming fewer non-steroidal anti-inflammatory drugs through reducing pain or prolonging the need for surgical intervention through assisting in disease self-management.

**Table 4:** Statements describing the potential contributions of nature prescribing to planetary health.

Section	#	Statement
**Planetary Health**		
*Nature prescribing can foster planetary health through…*		
	50	Connecting the human health and environmental disciplines (e.g., accepting concepts that might have been considered to be outside of a discipline, encouraging broader views)
	51	Being a cost-effective strategy to health management (e.g., chronic disease prevention, management of comorbid conditions)
	52	Including both human and ecological components
	53	Creating a positive feedback loop between human and environmental benefits
	54	Increasing eco-health literacy (local and global scales)
	55	Promoting nature stewardship (local and global scales)
	56	Simultaneously considering the health of people and nature
	57	Encouraging people to value nature more (e.g., increased care and awareness of the environment, improved emotional connection to nature)
*Specific to healthcare services, products, and systems, nature prescriptions can foster planetary health by…*		
	58	Reducing the carbon footprint of healthcare
	59	Reducing the environmental impact of healthcare production cycles (e.g., point of generation to disposal of treatment modalities)
	60	Substituting manufactured equipment with nature-based alternatives (e.g., using sand or uneven ground instead of foam pads to strengthen ankle muscles)
	61	Reducing the demand on ecologically damaging treatment options (e.g., surgical procedures, NSAIDs)
	62	Being an environmentally friendly intervention (e.g., provision of nature prescriptions as part of low-carbon healthcare)

*Note.* NSAIDs = Nonsteroidal anti-inflammatory drugs.

### Future directions: Research, education, and clinical practice

Future directions describes how the previous sections of the framework can be implemented in education and clinical practice, and provides recommendations for research (Table 5). We provide suggestions of where information about nature prescriptions should be implemented in healthcare education, from entry-level clinical education (Statement 86), to continuing education (Statement 90) and graduate programs (Statement 91), as well as further professional development for clinicians (Statement 92). Additionally, we suggest what information about nature prescriptions should be included in clinical education. Some suggestions attend to social and cultural considerations, such as information about Indigenous or cultural knowledge of nature and healing (Statement 96) and equity and access issues (Statement 104). Other suggestions are directed towards use in practice, including, but not limited to, guidance on dosage (Statement 97), the biopsychosocial benefits of being in nature (Statement 98), and the place of nature prescriptions in the broader context of healthcare (Statement 105). Fourteen research areas were recommended for future investigation, and nine knowledge translation formats to share information about nature prescriptions to potential knowledge end users. Lastly, clinical practice provides suggestions of how nature prescribing might be implemented and what should be included in nature prescriptions when used in healthcare practice. Suggestions are provided on the types of information that should be received by providers to inform nature prescribing, as well as the types of information patients or clients should know and receive to inform their use of nature prescriptions. Of note, the suggestions should be in accordance with the principles of person-centred care, and the prescribed nature-based activities should align with patient or client expectations and values.

**Table 5:** Recommendations for future directions of nature prescription research, education, and clinical practice.

Section	#	Statement
**Future Directions: Research**		
*Future research on nature prescriptions should include…*		
	63	Characteristics of professional contributions to nature prescribing (e.g., skills and/or training that aid nature prescription provision, existing link workers, necessary credentials and/or education)
	64	Feasibility of nature prescriptions in various contexts
	65	Barriers and facilitators to uptake (clinicians, patients/clients)
	66	Standard vs. flexible patient-centred nature prescriptions
	67	Different nature prescription activities (e.g., outdoor physical activity, gardening, indoor nature experiences)
	68	Ecosystem conservation and/or restoration activities as part of nature prescriptions
	69	Effects on health conditions and symptoms
	70	Interdisciplinary research to diversify evidence
	71	Impact and compliance of culturally relevant nature prescriptions
	72	Honouring Indigenous knowledges and wisdom (e.g., Land-based healing)
	73	Equity of nature prescriptions (e.g., distribution, access)
	74	Ownership and professionalization of nature (e.g., nature as belonging to the commons, nature-based therapies provided by nature vs. healthcare professionals)
	75	Adherence to nature prescription (e.g., ways to improve, factors affecting adherence)^a^
	76	Behaviour change and nature prescription (e.g., ways to incentivise, effectiveness)^a^
*Knowledge about nature prescriptions should be shared through…*		
	77	Infographics
	78	Inclusively designed art-based formats (e.g., songs, art exhibits)
	79	Social media (e.g., TikTok, Instagram, X)
	80	Technology-based formats (e.g., virtual and/or augmented reality, artificial intelligence)
	81	A dedicated nature prescription app
	82	Peer-reviewed journal articles
	83	Experiential learning opportunities
	84	Web-based formats (e.g., websites, blog posts)
	85	Conference presentations
**Future Directions: Education**		
*Information about nature prescribing should be included in…*		
	86	Entry-level clinical education
	87	Public health courses
	88	Clinical courses
	89	Exercise prescription courses
	90	Continuing education (e.g., courses, programs, CEUs)
	91	Graduate programs
	92	Professional development for clinicians
	93	Educational webinars
	94	Teaching on geography and nature^a^
*Education on nature prescribing should include…*		
	95	Experiential learning (e.g., participating as a recipient, practice prescribing to others, practice identifying community programs)
	96	Indigenous and/or cultural knowledge of nature and healing
	97	Guidance on dosage (e.g., frequency, intensity, duration, timing)
	98	Biopsychosocial benefits of being in nature (e.g., reduced pain, improved mental health)
	99	Risk mitigation strategies
	100	Potential nature activities to prescribe
	101	Common context-specific barriers
	102	Common context-specific facilitators
	103	Both research theories and evidence on nature and healing
	104	Equity and access issues
	105	The place of nature prescriptions in the broader context of healthcare and health interventions
	106	Information on planetary health to ground and contextualise nature prescription^a^
**Future Directions: Clinical Practice**		
*Nature prescriptions should…*		
	107	Be offered as an option to patients/clients with comorbid conditions for multiple gains where evidence supports its use
	108	Be tailored to target specific conditions (e.g., uneven ground can assist rehabilitation of ankle sprains)
	109	Be implemented to assist the management of chronic health conditions by attending to biopsychosocial factors
	110	Be used in all stages of healthcare provision, where evidence supports its inclusion (e.g., primary, secondary, and tertiary care provision)
	111	Be implemented at a stage personalised to each patient/client
	112	Be implemented at a stage that is consistent with an evidence-based approach (considering current evidence, patient views, and clinician experience)
	113	Be tailored to aid the prevention of specific conditions
	114	Be tailored to aid the management of specific conditions
	115	Be used as part of symptom management
	116	Be recorded in patient/client notes
	117	Be included in follow-up, where appropriate
	118	Incorporate fun activities
	119	Incorporate play-based activities, where appropriate
	120	Be a standard offering within usual practice for anyone who finds them valuable
	121	Include indoor nature connection experiences, where appropriate (e.g., live plants in clinics, nature videos while exercising)
	122	Include outdoor healthcare appointments when possible (e.g., outdoor physical therapy sessions)
	123	Include group-based outdoor programs, where appropriate
	124	Be used to address the biopsychosocial aspects of living with health conditions
	125	Align with functional activity goals (e.g., using gardening as a rehabilitation activity for a patient/client who previously enjoyed gardening)^a^
*Providers should receive information on…*		
	126	Ecosystem co-benefits of nature prescription (e.g., active transport to increase physical activity and reduce motor vehicle emissions)
	127	Biopsychosocial factors, benefits, and risks of nature prescription
	128	Locally available activities and programs (e.g., characteristics, location, cost)
	129	Existence of link workers to connect patients/clients with nature spaces
	130	Health conditions that nature prescriptions can help
	131	Guidance on how to prescribe nature (e.g., dosage, types of activities)
	132	Reasoning models to assist in shared decision making
	133	Insurance coverage regarding nature prescriptions
	134	Safety and privacy of nature prescriptions
	135	Strategies to reduce risk (for both people and nature)
*Information categories that patients/clients should know and receive to inform their use of nature prescriptions*		
	136	Patients/clients should be provided the same information about nature prescriptions as their provider
	137	Nature prescriptions should be co-designed to identify the information needs of individual patients/clients
	138	Information must be accessible and understandable to patients/clients (e.g., available in various formats and languages)^a^
*Patients/clients should receive information on…*		
	139	Local resources (e.g., parks, trails, outdoor programs, community resources)
	140	Safety of local nature spaces
	141	Research evidence in understandable formats
	142	Steps to take to experience their nature prescription (e.g., instructions, where to find additional resources and information)
	143	Constructive examples of what activities to do as part of their nature prescription
	144	Health benefits of nature prescriptions (general and condition specific)
	145	Potential risks and risk mitigation strategies
	146	Environmental benefits of nature prescribing

*Note.* CEUs = Continuing Education Units.

Link workers are non-health or social care professionals who have extensive knowledge of community resources and may have training in behaviour change or coaching [72]. They support people accessing local services in their community to improve health and well-being [72].

^a^  = Statement suggested as an addition during Round 2.

## Discussion

This study generated a consensus-based descriptive framework for nature prescribing in healthcare, including the potential impacts on people and the planet, integration of sociocultural considerations, role in planetary health, and guidance on how research, education, and clinical practice could proceed. Developed through interdisciplinary collaboration, a high-level shared understanding can provide a meta-framework of nature-based interventions in health and their use in healthcare whilst disciplinary theoretical or explanatory frameworks and causal pathways are refined in future work [51,52]. To address the need, we offer a framework developed by an interdisciplinary panel of experts to support the use of nature prescribing in clinical practice, complementary to existing frameworks and models [39,73–76] on nature prescribing and the human-environment-health nexus. We sought to propose a shared definition of nature prescription and a descriptive framework to guide the use of nature prescribing in healthcare, as well as answer two broad research questions: (1) How can the utilization of nature prescriptions address personal, social, environmental, and global burdens of health and healthcare? and (2) What is the direction for future research on and utilization of nature prescriptions?

Across multiple disciplinary fields, diverse assessments, models, and frameworks are currently being developed to enhance our understanding of the intricate relationships between people and the planet. In the recent Intergovernmental Science-Policy Platform on Biodiversity and Ecosystem Services Transformative Change Assessment, disconnection of people from nature and domination over nature and other people was identified as one of the leading causes of biodiversity loss [77]. The nature-based biopsychosocial resilience theory, a conceptual framework on nature-health relations, outlines how contact with nature can build and maintain biopsychosocial resilience-related resources to prevent, respond, and recover from stressors [74]. The International Classification of Functioning, Disability, and Health model describes environmental factors as the physical, social, and attitudinal environments that are essential to understanding a person’s experience of function and disability [76]. Similarly, the Person-Environment-Occupation-Performance Model describes the relationship between a person, their physical, psychological, social, and sensory environments, and how these factors can facilitate or limit engagement in activity and experiences of health and well-being [75]. A recent nature prescribing framework for Australian healthcare offers a structured approach to integrating nature prescribing through five essential domains: Community; Systems; Prescribers; Providing prescriptions; and External settings [39]. Our proposed framework complements existing works through providing a high-level synthesis of what interdisciplinary experts currently know and their collective judgement of nature prescribing, including a shared definition, potential benefits and risks, considerations, and suggestions for future research, education, and practice. As the body of research evidence continues to develop, the framework can serve as a blueprint for new findings to be incorporated and for the refinement of context-specific variants.

The framework statements provide a comprehensive overview of the benefits and risks of nature prescriptions, but do not intend to serve as best practice guidelines for both human and environmental health. Nuance and context must be appreciated when understanding, evaluating, and implementing the framework for planetary health benefits. If nature prescriptions are activity- or event-based, rather than merely spending time in nature, the risks to people and nature may be more direct through their interactions with nature spaces, such as vegetation trampling, congestion and crowding, litter, and pollution [78]. The environment-health co-benefits may be more direct when nature prescriptions include ecosystem conservation or restoration activities versus indirect co-benefits of increased greenspace use, such as increased funding for parks and promoting naturalised areas [30]. The increased human interaction with nature may pose a risk to the environment if additional greenspaces are developed and destroy the natural environment in the process, such as the degradation of ecosystems and forests to develop recreational spaces without considering ecosystem service flow, social contexts, and usage of human-developed greenspaces [79]. The inclusion of activities that support the health of nature, whilst meaning well, can cause harm. To reduce the risk, seeking input from a botanist or ecologist can help avoid causing unintentional harm [80]. The potential risks are more prominent when nature prescriptions are not advised or recommended properly. Exposure to nature could have more risks when the recipient does not understand when or how to engage with nature in a safe and relatively low risk way, such as going out when weather is good and not during a storm. The framework notes the planetary health potential of nature prescribing within healthcare systems and health services, such as reducing the carbon footprint of healthcare and reducing use of environmentally extractive health interventions. Engaging with nature through nature prescribing has several promising co-benefits for people and the environment, including enhanced pro-ecological behaviours and environmental stewardship of those participating [30], as well as promoting primary prevention and health promotion strategies that support environmentally sustainable care consumption [5]. However, the potential for increased degradation of natural areas with using nature as a context for health interventions may cause carbon emissions through changes to plant communities (e.g., die back) and soil ecosystems (e.g., increased carbon efflux). This juxtaposition of nature prescribing having the potential to both benefit and harm people and the planet should be highlighted. Without clear identification of such tensions, it is easy to be blinded by the promise of nature prescriptions without properly considering the potential costs. This finding aligns closely with notions of reciprocity and responsibility rooted in Indigenous Land- and country-specific First Laws [70,81]. This respect for, and recognition of relational and earth-centred systems [44], could bolster approaches to nature prescribing which support the well-being of humans and nature.

The proposed framework highlights the influence of social and cultural contexts on inclinations towards nature, such as how nature is defined, the health and environmental impacts of nature prescriptions, and equitable person-centred and culturally competent care. There is a need for a definition of nature prescriptions beyond the traditional Western notion of a prescription pad to recognise and support other sociocultural contexts, including oral and spoken approaches that amplify Indigenous knowledges of Land-based healing [71]. Thus, the definition of nature prescriptions provided by this framework allows for adaptation to resonate with diverse sociocultural contexts, worldviews, and knowledge. The framework statements are to serve as a guide subject to change depending on context to ensure culturally competent and person-centred care, such as considering contextual factors of geographic, systemic, and institutional discrimination and racism [82,83]. When cultural competency and humility are upheld by healthcare professionals [84,85], nature prescriptions may provide physical, mental, social, and spiritual benefits to marginalised groups. Through clinical and contextual judgement, nature prescription providers can ensure equitable access to the benefits of nature and reduce the risk of harm or discomfort.

### Strengths and limitations

A key strength of this modified Delphi study is the interdisciplinary expertise of the panel participants recruited from three continents. Previous literature on Delphi methodology has suggested that a heterogeneous and diverse expert panel allows for a wider range of perspectives and knowledge, leading to better performance [55] and more reliable responses [55,86]. However, a key limitation is the absence of Indigenous expertise from the panel participants, which shapes the applicability of findings and is an important area of consideration for future studies. An additional limitation related to representation is only including those who could participate in English. The non-anonymous nature of data collection allowed for strong rapport between the lead researcher (NS) and members of the expert panel, as well as between panellists, and facilitated active engagement and iterative discussions. The co-construction of knowledge by panellists from varying disciplines with expertise related to nature prescription and health resulted in the development of a robust, preliminary framework. Furthermore, co-construction was characterised by numerous rich engagements amongst expert panellists, contributing to the interdisciplinarity of statement development and the connections within and amongst themes. Lastly, resulting from the characteristics of how “nature” and “prescription” were understood, we offer a definition of nature prescription that was informed by the cumulative learning experiences and expertise of the expert panel. The lack of a shared definition of nature prescription has been identified as a constraint to implementation in healthcare and research [29,49,50]; thus, a strength of this study is the provision of an interdisciplinary definition that can be used in various research contexts and professions.

A limitation of this study is its smaller sample size, likely contributed to by the high level of participant engagement and time commitment, in addition to research on nature prescription being in its infancy with relatively few experts to recruit from. Despite this, the final sample size of n = 8 aligns with recommendations for eight panellists as the minimum sample size for Delphi studies [87,88], and was appropriately representative. Delphi group sizes do not rely on statistical power, thus, group dynamics are of higher importance than sample size for a Delphi expert panel [54]. Focus groups did not allow for anonymity of panellists, which has been recognised as an important feature of Delphi studies to reduce dominating voices [54]. The expert panel was largely from the Global North and identified as female. Representation of perspectives and worldviews from the Global South and non-Western systems was limited; thus, affecting the applicability and transferability of the framework. Although Indigenous knowledge was central to the conceptual foundation of this framework, no Indigenous participants were included in the expert panel. Future studies would benefit from deliberate inclusion of Indigenous voices both as co-researchers and co-developers. While the expert panel provided strong interdisciplinary representation, its limited geographic and cultural diversity may constrain the global applicability and interpretive breadth of the framework. Considering the cultural relativity of nature, healing, and prescription, future research should aim to include experts and communities across diverse socio-ecological and cultural systems, particularly from the Global South and Indigenous contexts. Accommodating for different time zones did influence the group sizes and demographics represented within each focus group; thus, group dynamics could have affected open-ended responses and the overall discussions. The small sample size and lack of diversity of the expert panel limit the transferability of the results of this study, and judgement by the reader should be held to determine the applicability of the framework to diverse contexts. Considering these geographic and sociocultural limitations of this study, future research should seek to hold these discussions in contexts with competing human health and development needs. The two-round modified Delphi process resulted in a total of 146 framework statements. Subsequent rounds may have further refined and reduced the size of the framework by omitting statements of less importance; however, the framework is described by central themes which helps reduce the quantity of information that must be processed when using the framework.

### Recommendations for future research

We propose recommendations for future research (Table 5: Statements 84–97) underlining the importance of interdisciplinarity throughout the research process and diversification of the evidence supporting nature prescribing. This has potential to enhance transdisciplinary findings, develop collaborations between fields, and encourage research leadership between the health and environment sectors. Future research should consider evaluating patterns of agreement and disagreement, who agrees or disagrees, and the reasons for disagreement. To support the uptake of nature prescriptions in healthcare, the body of evidence on health effects, barriers and facilitators to implementation and uptake, as well as adherence and compliance should be expanded. Providing clinicians the necessary evidence to inform the decision-making process may facilitate the integration of nature prescribing into evidence-based practice as an intervention aiding the progression towards sustainable healthcare whilst offering biopsychosocial benefits. The impact of nature prescriptions on pro-environmental behaviours, human and environmental co-benefits, engagement with greenspaces and nature, and biodiversity should be further explored, with the goal of better understanding the environmental impact of nature prescribing. Investigating the perspectives of general and clinical populations, including how they conceptualise nature prescriptions, may reveal a limited degree of shared understanding which could have implications on uptake if research and practice perspectives are divergent. The framework presented here is based on the knowledge, judgements, and opinions of experts; however, further research is needed to validate the framework content if best practice guidelines for nature prescribing are to be developed. Clinical disciplines should draw on their clinical practice guidelines and best available evidence to adapt this framework to their scope of practice, patient population, and geographic and contextual factors. Future research, including adaptations of the proposed framework for different health disciplines and clinical settings, should seek to improve practical utility and consider both systems and discipline-specific perspectives. The representation of the panel participants may not have adequately captured the healthcare systems perspective, as nature prescription could also be useful to counsellors, psychologists, psychiatrists, and behavioural medicine experts, amongst other clinical disciplines.

## Conclusion

This study presents a new framework for the use of nature prescribing in healthcare, synthesised from the cumulative experience and expertise of leaders and experts in nature prescription. Nature prescribing can benefit people and the planet when the principle of do no harm is upheld alongside person-centred culturally competent care. To address burdens of health and healthcare, consideration should be given to the benefits and risks of nature prescription, potential planetary health impacts, and how nature prescribing can be equity-driven by incorporating social and cultural differences. Our findings provide direction and recommendations for future research, education, and clinical practice regarding nature prescribing as an intervention for people and the planet, as well as its place in the broader context of healthcare. Sustainable healthcare is increasingly important as ongoing anthropogenic climate change continues to threaten the health of people and our planet. Moving forward, nature prescription may be a constituent of global actions to achieve planetary health.

## Supporting information

S1 Table Demographic characteristics of the expert panel participants. (DOCX)

S1 File Statement % (95%CI) & Statement Modifications %. (XLSX)
