# Supplementary material for: An international and interdisciplinary framework for nature prescribing in healthcare: A modified Delphi study
Source: PLOS Glob Public Health. 2026 Apr 30;6(4):e0006361. doi: 10.1371/journal.pgph.0006361 (PMC13132205; doi:10.1371/journal.pgph.0006361)
Supplement: S1 Table — (DOCX) [file pgph.0006361.s001.docx]

**Supporting Information**

**Demographic Characteristics of the Expert Panel**

A total of 30 experts were identified and 24 who met the inclusion criteria were invited to participate in the study. Of them, 11 consented to participation, eight completed both Round 1 and Round 2, and three participants were lost to follow-up.

**S1 Table**

Demographic characteristics of the expert panel participants.

| Variable | Recruited  N =11 (%) | Completed  N = 8 (%) |
| --- | --- | --- |
| **Sex, N (%)** |  |  |
| Male | 2 (18) | 2 (25) |
| Female | 9 (82) | 6 (75) |
| **Age, N (%)** |  |  |
| 21-30 years of age | 1 (9) | 1 (13) |
| 31-40 years of age | 2 (18) | 2 (25) |
| 41-50 years of age | 6 (55) | 4 (50) |
| 51-60 years of age | 1 (9) | 0 (0) |
| 60+ years of age | 1 (9) | 1 (13) |
| **Country of residence, N (%)** |  |  |
| Australia | 2 (18) | 2 (25) |
| Canada | 5 (46) | 3 (38) |
| Norway | 1 (9) | 1 (13) |
| Sweden | 1 (9) | 0 (0) |
| USA | 2 (18) | 2 (25) |
| **Highest degree level, N (%)** |  |  |
| Bachelor's degree | 0 (0) | 0 (0) |
| Master's degree | 1 (9) | 1 (13) |
| Doctoral degree (e.g., PhD, DPhil) | 8 (73) | 6 (75) |
| Professional degree (e.g., Medicine MD, Law JD) | 2 (18) | 1 (13) |
| **Population centre size, N (%)** |  |  |
| Between 1,000 and 29,999 | 1 (9) | 1 (13) |
| Between 30,000 and 99,999 | 2 (18) | 2 (25) |
| 100,000 and over | 8 (73) | 5 (63) |
